# Holographic hyperbranched polymer nanocomposite grating with exceptionally large neutron scattering length density modulation amplitudes

**DOI:** 10.1038/s41598-025-16998-z

**Published:** 2025-08-26

**Authors:** Elhoucine Hadden, Jürgen Klepp, Martin Fally, Tobias Jenke, Joachim Kohlbrecher, Tomoko Shimada, Asako Narita, Juro Oshima, Yasuo Tomita

**Affiliations:** 1 Faculty of Physics & Vienna Doctoral School in Physics, University of Vienna, 1090 Vienna, Austria; 2 Institut Laue-Langevin, 71 avenue des Martyrs, CS 20156, 38042 Grenoble Cedex 9, France; 3 Faculty of Physics, University of Vienna, 1090 Vienna, Austria; 4 PSI Center for Neutron and Muon Sciences, 5232 Villigen PSI, Switzerland; 5 Department of Engineering Science, University of Electro-Communications, 1-5-1 Chofugaoka, Chofu, Tokyo 182-8585 Japan; 6 Materials Research Laboratories, Nissan Chemical Corporation, 488-6 Suzumi, Funabashi, Chiba, 274-0052 Japan; 7 Present Address: Department of Physics and Astronomy, Uppsala University, Box 516, 75120 Uppsala, Sweden

**Keywords:** Materials science, Nanoscience and technology, Optics and photonics, Physics

## Abstract

Nanoparticle–polymer composite gratings incorporating ultrahigh-refractive-index hyperbranched polymers as organic nanoparticles have demonstrated exceptional light optical properties, yet their potential for neutron diffraction applications remains unexplored. We report on the neutron optical properties of a holographically structured hyperbranched-polymer–dispersed nanocomposite grating at a quasi-monochromatic neutron wavelength of 2 nm. We show that neutron diffraction measurements performed at the SANS-I instrument of the Paul Scherrer Institute (Switzerland) reveal exceptionally high neutron scattering length density modulation amplitudes. These scattering length density modulation amplitudes are the highest reported to date. Very high neutron diffraction efficiency is expected with the use of thicker uniform gratings and longer neutron wavelengths, with low angular and wavelength selectivity constraints.

## Introduction

Neutron interferometry is a uniquely sensitive technique for probing both material properties and fundamental quantum phenomena^1^. Its ability to detect coherent phase shifts enables precision measurements of scattering length densities (SLDs)^2^ and has supported landmark experiments, including the demonstration of $4\pi$-symmetry of fermionic wavefunctions, gravitational quantum interference, and neutron spin-path entanglement^3^. Extending neutron interferometry into the cold neutron (CN) and very cold neutron (VCN) wavelength regimes enhances sensitivity to quantum effects. A first generation of VCN interferometers has been operated since the end of the 1980 s, notably with the establishment of a dedicated platform at the PF2/VCN instrument at the Institut Laue Langevin (ILL)^4^. Despite the scientific interest in such experiments, the boom has gradually declined due to two main limitations: the inherently low flux of VCN beams and the lack of efficient neutron-optical components adapted to longer neutron wavelengths. Addressing the need for wavelength-adapted optical elements is crucial to enable the next generation of highly sensitive slow neutron interferometers.

Regarding neutron source limitations, the development of advanced neutron facilities is underway, incorporating dedicated VCN sources to overcome flux constraints^5^. For instance, the HighNESS project aims to establish a high-flux VCN source at the European Spallation Source (ESS), promising substantial improvements in available neutron flux^6^. However, fully exploiting these advances requires complementary progress in neutron optical components. Traditional neutron optics for interferometry such as perfect single-crystals suffer from intrinsic limitations at VCN wavelengths. An alternative approach is the use of holographic optical elements by transforming blends of photosensitive materials into artificial periodic structures. Several material classes along this line have been reported so far. These include gratings recorded in thick binder-based PMMA photopolymer^7^, holographic polymer-dispersed liquid crystals (HPDLCs)^8^, and nanoparticle polymer composites (NPCs)^9,10^. It was found that the 1 mm-order thickness of the PMMA gratings resulted in low neutron diffraction efficiency due to the Pendellösung interference effect^9^. HPDLCs were also found to suffer from high anisotropy causing significant light scattering during recording and thereby resulting in low neutron diffraction efficiency. On the other hand, NPCs, which were originally developed for holographic gratings with high diffraction efficiency at visible wavelengths, low polymerization shrinkage, and high thermal stability—desirable for holographic data storage, nonlinear optics, and wearable displays^9^—have also been found efficient at slow neutron wavelengths. It was also found recently that the use of nanodiamonds, with their large SLD value, as nanoparticles in NPCs provided high performance in neutron diffraction^11–13^.

In this work, we describe an experimental investigation of neutron diffraction properties of a holographic NPC grating incorporating hyperbranched polymer (HBP) as organic nanoparticles—originally developed for other photonic applications^14–16^—at a mean neutron wavelength of 2 nm.

## Materials and methods

**Fig. 1:**
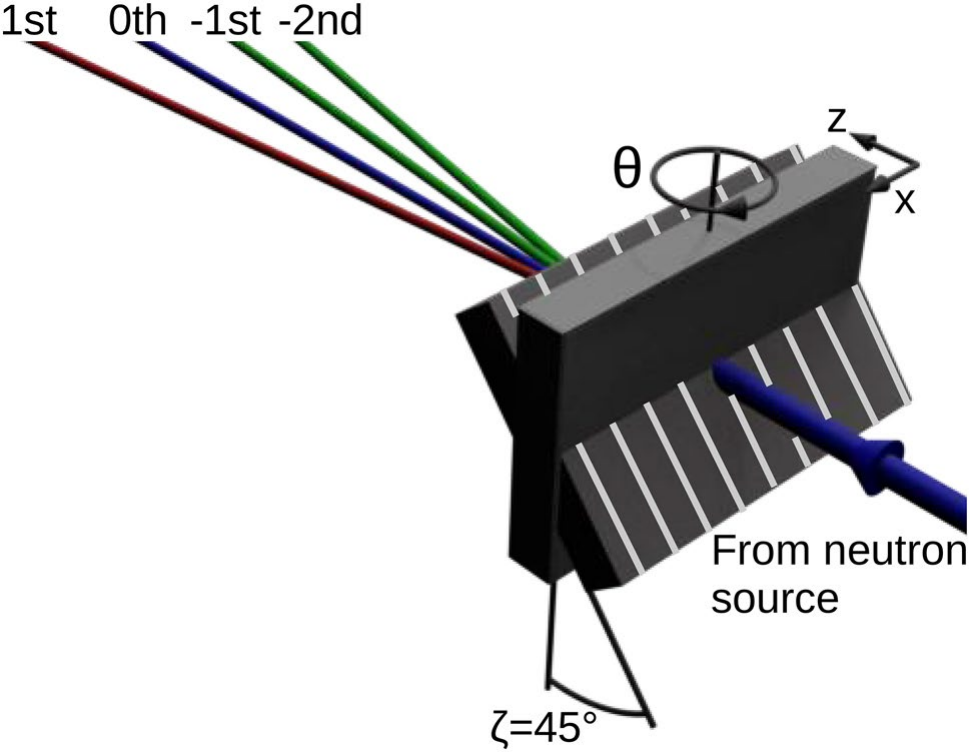
Illustration of the experimental geometry for neutron diffraction from an unslanted holographic phase grating in transmission mode. Diffraction is measured as a function of stepwise rotation of the sample by an angle $\theta$ around the *y* axis. Tilting the sample by an angle $\zeta < 90^\circ$ around the grating vector (aligned with the *x* axis) increases the effective NPC film thickness (along the *z* axis) from its physical value *d* to $d (\zeta ) = d (0) /\cos \zeta$.

Full details of the synthesis, chemical structures and optimization study for red-light recording are provided in Ref.^16^ and its supplement^17^. Here, we provide a brief description of the considered grating. Hyperbranched polymers were synthesized from m-phenylenediamine (m-PDA), TCT: 2,4,6-Tris(trichloromethyl)−1,3,5-triazine and N,N-dimethylacetamide (DMAc). The resulting molecular structure shows aromatic ring units ensuring thermal and mechanical stability, a key feature for application in neutron interferometry. The organic nanoparticles have an ultrahigh refractive index $n_{\text {NP}} = 1.82$ compared to the host polymer $n_{\text {P}} = 1.50$^14^. The NPC films were formed by dispersing HBP powder at 25 vol.% in a mixture of the host monomer 4-hydroxybutyl acrylate (72 vol.%) and the crosslinker A-DPH-12E (3 vol.%). Recording was enabled by a three-component photosensitizer–initiator system consisting of cyanine dye: 3, 3’-Dipropylthiadicarbocyanine Iodide $\text {DiSC}_3 \text {(5)}$, TCT (electron acceptor), and a borate salt (N3B, electron donor). The study of fluorescence quenching, polymerization and buildup dynamics in Ref.^16^ demonstrates that the employed relative molar concentration 1:10:8 of $\text {DiSC}_3 \text {(5)}$:TCT:N3B combines efficient radical generation with a delayed gelation process, facilitating mutual diffusion of nanoparticles and monomer molecules during the holographic assembly. Moreover, the HBP percentage of 25 vol.% results in the highest achievable refractive index modulation amplitude. Further increasing this value results in an exponential rise in viscosity. The recording process of an unslanted holographic phase grating employed a conventional two-wave mixing setup and a single-longitudinal mode laser operating at 640 nm. This results in a spatially modulated refractive index pattern $n(x, z) = n_0 + \sum _{j> 0} n_j(z) \cos {(j G x + \varphi _j)}$. Here, $n_0$ is the averaged refractive index of the NPC film, $(G = 2 \pi / \Lambda )$ the modulus of the grating vector with $\Lambda = 500$ nm being the grating period, $n_j$ are the Fourier components (FCs) of the refractive index modulation, and $\varphi _j$ the corresponding relative phases. Diffraction occurs in transmission mode for light and for neutrons. In both cases, the sample was mounted on a rotational stage to enable the construction of rocking curves through angular $\theta$-scans around normal incidence. For neutrons, it was also tilted at a fixed angle $\zeta = 45^\circ$ around the grating vector as illustrated in Fig. 1, in order to increase the effective thickness for diffraction (see, for instance, Ref^10^.).

## Results and discussion

### Light-optical diffraction study

**Fig. 2:**
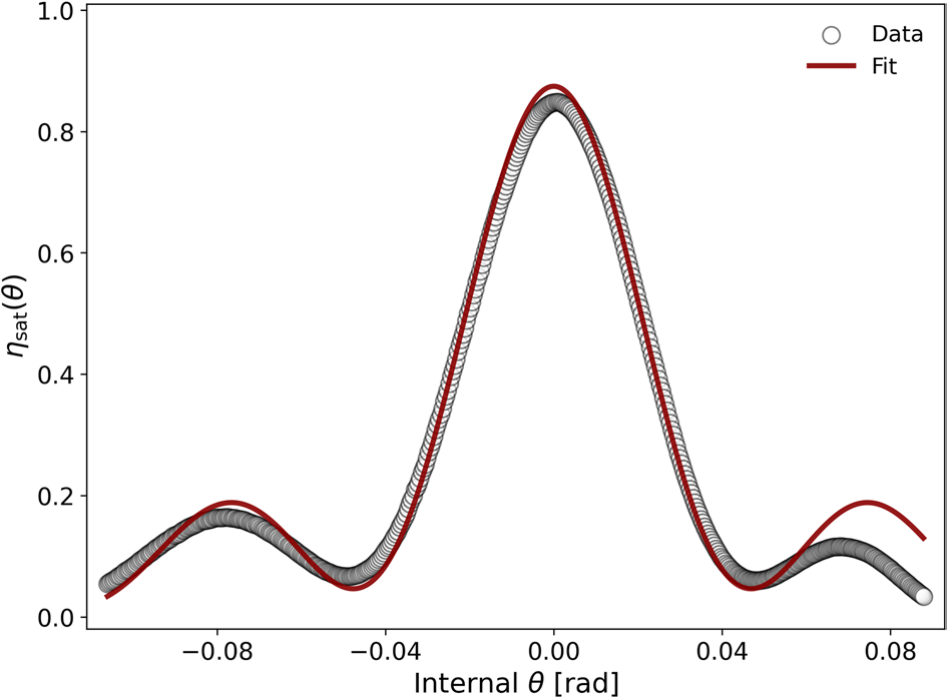
Bragg-angle detuning curve of $\eta _{\tiny {\text {sat}}}$ probed at 532 nm (empty circles), together with the model fit from standard least-squares minimization (solid line).

The angular selectivity of the saturated diffraction efficiency $\eta _{\tiny {\text {sat}}}(\theta )$, where $\theta$ is the Bragg-angle detuning-was measured by a readout laser beam at 532 nm. The corresponding curve is shown in Fig. 2.

The observed asymmetry in the data is attributed to secondary scattering, the origin of which will be explained in the subsequent analysis. In addition, the lifted side-lobe minima indicate a grating decay along the thickness direction $z$^14,15^. This non-uniformity can be explained by a thickness dependence of the first-order refractive index modulation amplitude $n_1 (z)$. Therefore, the analysis was performed with a least-squares fit to $\eta _{\tiny {\text {sat}}}(\theta )$ using Uchida’s formulation^18^. The latter considers an exponential attenuation profile of $n_1(z) = n_{10}\exp (-z/L)$, where $d$ is the physical film thickness and $L$ is the effective decay length of the recorded grating. The used model is based on the beta-value method (BVM) boundary conditions^19^. The best-fit parameters are a grating thickness of $d \approx 8.4\,\mu \textrm{m}$, a surface modulation amplitude $n_{10} = (5.6 \pm 0.0) \times 10^{-2}$, and a decay length $L \approx 9.3\,\mu \textrm{m}$. The comparable values of the attenuation length *L* and the thickness $d$, attributed to grating attenuation from holographic scattering during recording^15,20^ in a sample with extremely large $n_{10}$. The calculated effective grating thickness $d_{\textit{eff}}$, defined as $L \big [1 - \exp (-d/L) \big ]$, is $5.5\,\mu \textrm{m}$, and the thickness-averaged modulation amplitude $\langle n_1(z) \rangle$, defined as $n_{10} \, d_{\textit{eff}} / d$, is $(3.7 \pm 0.2) \times 10^{-2}$. Subsequently, the mutual diffusion during photopolymerization can be evaluated by calculating the modulation of the nanoparticle volume fraction, $a_1 \Delta f$, induced by the recording process^11^. Here, $a_1$ denotes the first-order Fourier component of a periodically modulated pattern (equal to unity for a purely sinusoidal modulation), while $\Delta f$ is the amplitude of the HBP nanoparticle volume fraction modulation within the Maxwell–Garnett approximation for multicomponent photopolymer systems. It is calculated via $a_1 \Delta f = \langle n_1 \rangle / |\text {n}_{\tiny {\text {NP}}} - \text {n}_{\tiny {\text {P}}}|$, yielding $a_1 \Delta f = 0.114$. This value is roughly 8 times higher than the best previously reported for nanodiamond-based NPC gratings^12,13^, which motivates the subsequent investigation of its neutron-optical performance.

### Neutron diffraction study

**Fig. 3:**
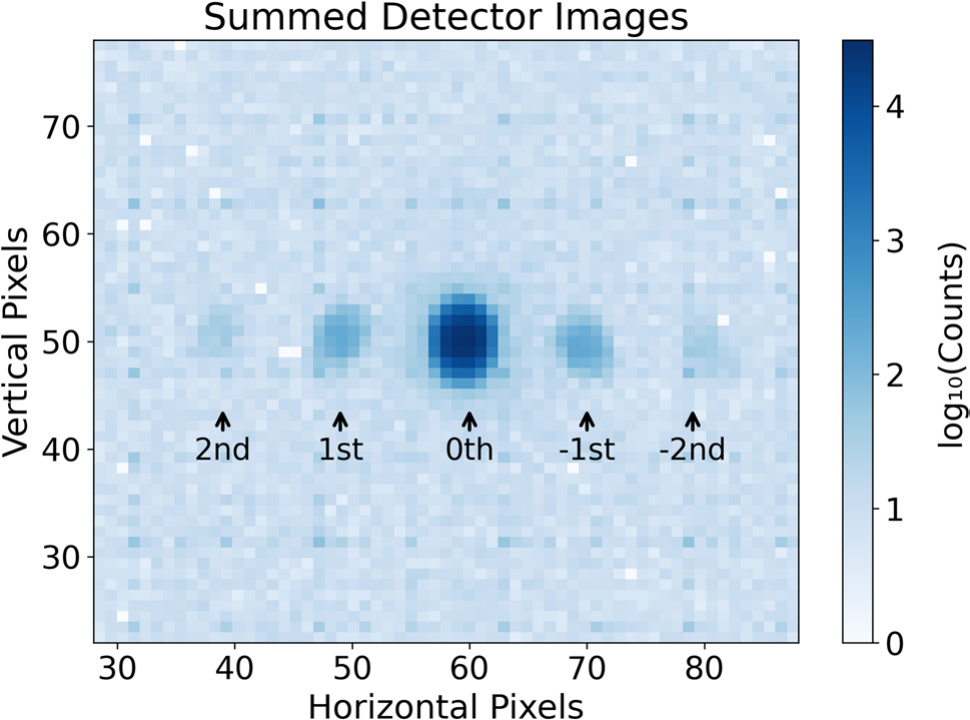
Accumulated detector image obtained by summing the recorded counts at each pixel over all angular positions of the $\theta$-scan, displayed on a logarithmic color scale of the total counts.

Diffraction measurements were performed at the SANS-I instrument at the Swiss Spallation Neutron Source (SINQ), Paul Scherrer Institute (PSI). A mean neutron wavelength of $\lambda =2$ nm was selected using a helical slot velocity selector, with a relative wavelength spread of $\Delta \lambda / \lambda \approx 0.1$. Beam collimation was achieved using two apertures separated by 18 m: a 30-mm aperture at the entrance and a 4-mm aperture behind the sample. The resulting beam divergence of about 1.9 mrad is much narrower than the expected diffraction peak width, approximated by $2\Lambda /d \approx 0.12$ rad, ensuring sufficiently high angular resolution. A two-dimensional $^3$He neutron detector with $128\times 128$ pixels of $7.5\times 7.5\,\hbox {mm}^2$ size is placed 18 m away from the sample and was used to record the diffracted intensities. The data collected during the $\theta$-scan were summed over all angular positions to construct the accumulated detector image shown in Fig. 3.

**Fig. 4:**
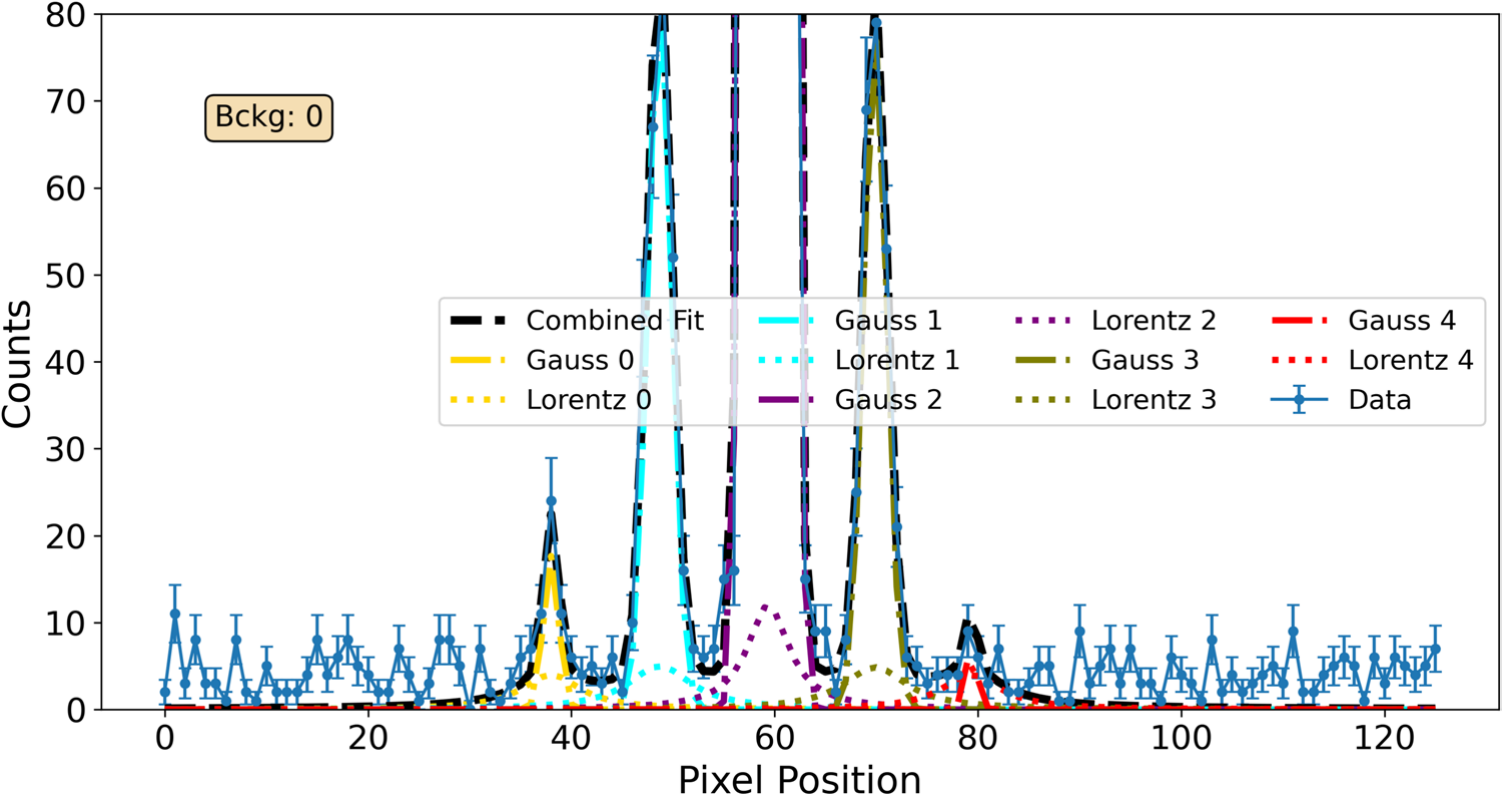
Horizontal profile fit with multiple peaks modeled as a sum of Gaussian and Lorentzian components on a constant background, shown at an arbitrary angular position of the rocking curve. Peaks are labeled 0–4 from left to right by their detector positions.

Diffraction spots up to the second order are clearly visible around the intense forward-diffracted (0th order) beam. A logarithmic scale is used to enhance the visibility of the weaker second-order peaks. Data were extracted and explored with Grasp software and custom Python scripts. For each diffraction spot, a bounding region of interest was defined, and counts from all runs at each angle were summed to obtain raw counts. The sufficiently wide angular span permitted background estimation from off-Bragg counts, defined as the minimum of the rocking-curve counts for each diffracted order (excluding the forward-diffracted order). For the latter, where this definition is ambiguous, a horizontal-profile fit was performed as shown in Fig. 4. The profile fit included a constant background (equal to zero in the example shown), with each order modeled as a combination of Gaussian (specular) and Lorentzian (diffuse background) components^21^.

**Fig. 5:**
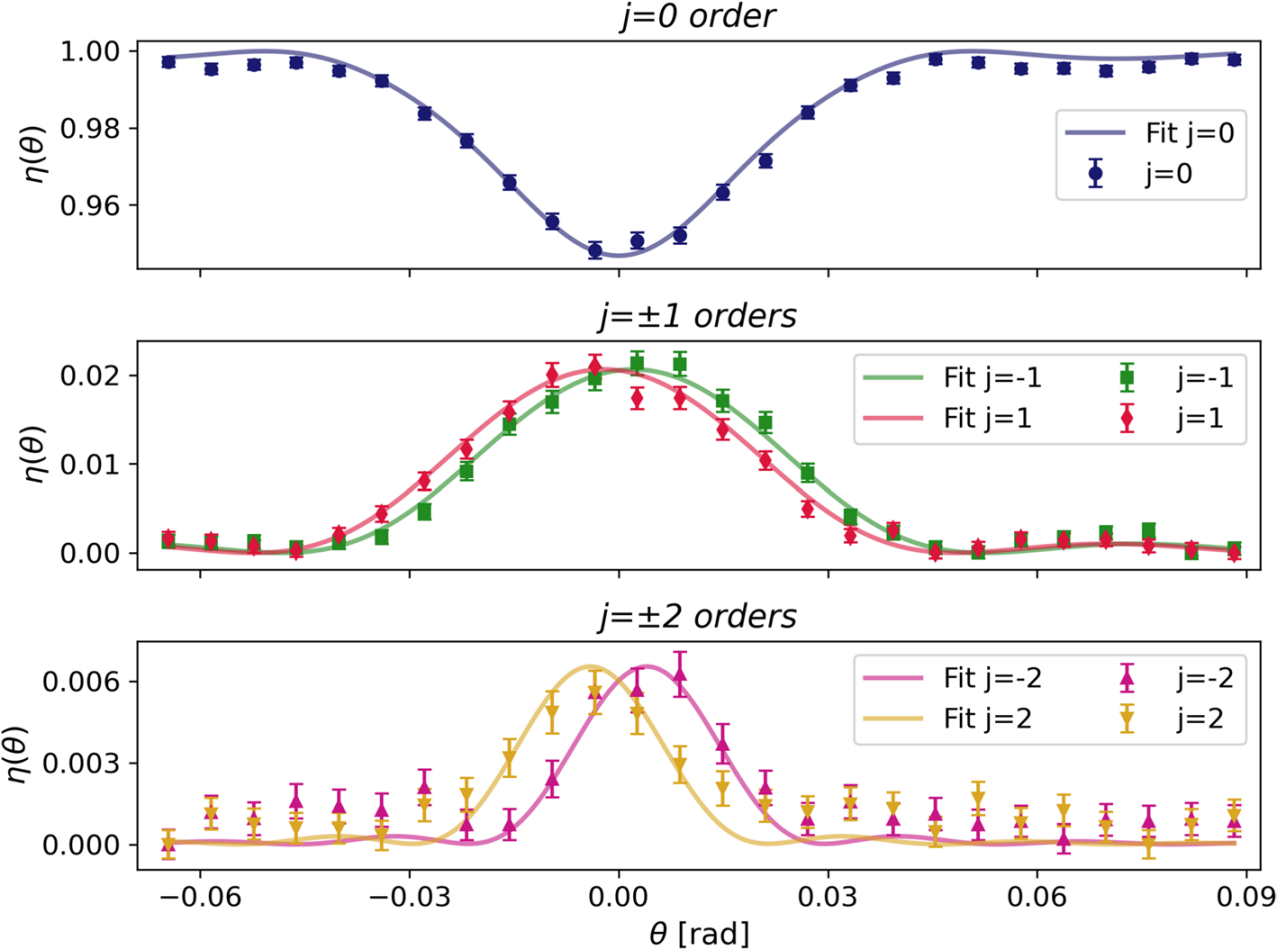
Neutron diffraction efficiency curves of the HBP-dispersed NPC grating (symbols) and corresponding fits (solid lines) at a neutron wavelength of 2 nm.

Full details, including background-spread analysis and comparison of background estimation methods, are provided in Ref.^22^. The resulting raw counts and background estimates were then used to compute diffraction efficiencies, with uncertainties obtained by standard error propagation. The corresponding curves along with their fits are presented in Fig. 5.

The efficiency curves were analyzed using a truncated first-order seven-coupled waves (7-CW) analysis with BVM boundary conditions^22,23^. The model accounts for the first- and second-order FCs of the SLD modulation. The SLD is defined as the product of the neutron coherent scattering length $b_c$ and the number density $\rho$, and relates to the neutron refractive index via: $n_{\text {N}} = \sqrt{1 - \frac{\lambda ^{2}}{\pi }\,(b_c \rho )}$. This approach is justified by the presence of significant diffraction into five orders ($j=0$, $\pm 1$, $\pm 2$). To account for all coupling terms up to the second diffraction orders, a 7-CW analysis is required. This is accomplished at reasonable computational costs. In the neutron diffraction analysis, the *j*-th SLD modulation amplitude $\Delta (b_c \rho )_j$ ($j = 1, 2$) is related to the corresponding neutron refractive index modulation amplitude $n_j$ by $\Delta (b_c \rho )_j = \frac{2\pi }{\lambda ^2} n_j$, with $|\Delta (b_c \rho )_j| = a_j \Delta f \, \left| (b_c \rho )_{\textrm{NP}} - (b_c \rho )_{\textrm{P}} \right|$^11^. The fit yields an extremely large first-order thickness-averaged SLD modulation amplitude, $\langle \Delta (b_c \rho )_1 \rangle = (14.9 \pm 0.2)$ $\mu \hbox {m}^{-2}$. This marks a net improvement over the highest averaged value of $(8.2 \pm 0.7)$ $\mu \hbox {m}^{-2}$ recently reported for nanodiamond-dispersed NPC gratings^13^. A comparable magnitude to the latter is found for the second-order component, $\langle \Delta (b_c \rho )_2 \rangle = (-8.2 \pm 0.3)$ $\mu \hbox {m}^{-2}$. The negative sign reflects a relative phase $\varphi _2 = \pi$ between the first- and second-order components of the HBP-dispersed NPC grating, and hence, resulting in the real part of the refractive index $n(x) \propto |<n_1>| \cos {(Gx)} - |<n_2>| \cos {(2Gx)} + ..$. The availability of reference beams in the multi-wave coupling allows for phase retrieval and reconstruction of the grating structure as described in Ref.^24^. The observed symmetric peaks exclude the possibility of $\pi / 2$ phase shifts between the FCs, a signature of dynamical holography. In most common cases, relative phases take one of the two values: 0 or $\pi$, determining whether diffraction orders mutually interfere constructively or destructively. Although the intrinsic SLD contrast of HBP nanoparticles in the host polymer is modest relative to inorganic-nanoparticle systems, the large value of the volume fraction modulation $a_1 \Delta f$ achieved here demonstrates that the considered organic nanoparticles migrate substantially more efficiently toward the dark regions of the interference pattern during holographic assembly. The high photosensitivity and favorable diffusion properties promote efficient phase separation. This enables SLD modulation amplitudes that exceed conventional limits. The first-order diffraction efficiency oscillations can be approximated as $\eta _{\pm 1}(\theta _B) \simeq \sin ^2{\left( \dfrac{\lambda d}{2} \Delta (b_c \rho )_1\right) }$. Exploring the Pendellösung effect with such high values, either by using a longer wavelength or by increasing the grating thickness, would allow mirror-like reflection without significant flux losses due to wavelength or angular selectivity in thick holograms.

## Conclusion

HBP-based NPC gratings represent promising candidates for neutron-optical applications in the cold and very cold neutron regimes. The material exhibits exceptionally high neutron SLD modulation amplitudes, surpassing previous benchmarks. While the current grating thickness limits diffraction efficiency at $\lambda =2$ nm, a moderate thickness increase is expected to substantially enhance the performance. Future research should address the non-trivial challenge of optimizing material composition and recording conditions to allow moderate increases in grating thickness while maintaining large modulation contrast and structural integrity. A previous study indicates that the inverse of the absorption constant at the recording wavelength $\lambda _{rec}=640$ nm is $72\,\upmu \hbox {m}$ before recording and $725\,\upmu \hbox {m}$ after, suggesting that the task is feasible. However, secondary scattering related to the high modulation amplitudes, which also scales with increasing thickness, causes the propagating interference pattern to deteriorate during recording, which makes it more intricate. These efforts will be essential for meeting the demands of next-generation CN and VCN instrumentation and for complementing ongoing advancements in high-flux neutron sources.

## Data Availability

Raw data were generated at the Paul Scherrer Institute large scale facility. Derived data supporting the findings of this study are available from the corresponding author upon reasonable request.

## References

[1] Rauch, H. & Werner, S. A. *Neutron Interferometry: Lessons in Experimental Quantum Mechanics, Wave-Particle Duality, and Entanglement* (Oxford University Press, 2015).

[2] Haun, R. et al. Precision measurement of the neutron scattering length of using neutron interferometry. *Phys. Rev. Lett.* **124**, 012501. 10.1103/PhysRevLett.124.012501 (2020). 10.1103/PhysRevLett.124.012501 PMC8609613 31976711

[3] Danner, A. et al. Three-path quantum cheshire cat observed in neutron interferometry. *Commun. Phys.* **7**, 14. 10.1038/s42005-023-01494-5 (2024).

[4] Eder, K. et al. The new very-cold-neutron optics facility at ILL. *Nucl. Instrum. Methods Phys. Res., Sect. A* **284**, 171–175. 10.1016/0168-9002(89)90273-8 (1989).

[5] Mezei, F. Very cold neutrons in condensed matter research. *J. Neutron Res.* **24**, 205–210 (2022) https://journals.sagepub.com/doi/full/10.3233/JNR-220012.

[6] Santoro, V. et al. The HighNESS project at the european spallation source: Current status and future perspectives. *Nucl. Sci. Eng.* **198**, 31–63. 10.1080/00295639.2023.2204184 (2024).

[7] Rupp, R. A., Hehmann, J., Matull, R. & Ibel, K. Neutron diffraction from photoinduced gratings in a PMMA matrix. *Phys. Rev. Lett.* **64**, 301–302. 10.1103/PhysRevLett.64.301 (1990). 10041945 10.1103/PhysRevLett.64.301

[8] Fally, M., Drevensek-Olenik, I., Ellabban, M., Pranzas, K. & Vollbrandt, J. Colossal light-induced refractive-index modulation for neutrons in holographic polymer-dispersed liquid crystals. *Phys. Rev. Lett.* **97**, 167803. 10.1103/PHYSREVLETT.97.167803 (2006). 17155436 10.1103/PhysRevLett.97.167803

[9] Tomita, Y. et al. Photopolymerizable nanocomposite photonic materials and their holographic applications in light and neutron optics. *J. Mod. Opt.* **63**, S1–S31. 10.1080/09500340.2016.1143534 (2016). 27594769 10.1080/09500340.2016.1143534 PMC4986931

[10] Fally, M. et al. Neutron optical beam splitter from holographically structured nanoparticle-polymer composites. *Phys. Rev. Lett.* **105**, 123904. 10.1103/PhysRevLett.105.123904 (2010). 20867643 10.1103/PhysRevLett.105.123904

[11] Tomita, Y. et al. Fabrication of nanodiamond-dispersed composite holographic gratings and their light and slow-neutron diffraction properties. *Phys. Rev. Applied* **14**, 044056. 10.1103/PhysRevApplied.14.044056 (2020).

[12] Hadden, E. et al. Nanodiamond-based nanoparticle-polymer composite gratings with extremely large neutron refractive index modulation. In *Photosensitive Materials and Their Applications II*, vol. 12151, 70–76, 10.1117/12.2623661 (SPIE, 2022).

[13] Hadden, E. et al. Holographic nanodiamond polymer composite grating with unprecedented slow-neutron refractive index modulation amplitude. *Appl. Phys. Lett.* **124**, 071901. 10.1063/5.0186753 (2024).

[14] Tomita, Y. et al. Nanoparticle-polymer composite volume holographic gratings dispersed with ultrahigh-refractive-index hyperbranched polymer as organic nanoparticles. *Opt. Lett.* **41**, 1281–1284. 10.1364/OL.41.001281 (2016). 26977689 10.1364/OL.41.001281

[15] Tomita, Y. et al. Very high contrast volume holographic gratings recorded in photopolymerizable nanocomposite materials. *Opt. Express* **28**, 28366–28382. 10.1364/OE.400092 (2020). 32988109 10.1364/OE.400092

[16] Narita, A., Oshima, J., Iso, Y., Hasegawa, S. & Tomita, Y. Red-sensitive organic nanoparticle-polymer composite materials for volume holographic gratings with large refractive index modulation amplitudes. *Opt. Mater. Express* **11**, 614–628. 10.1364/OME.415422 (2021).

[17] Narita, A., Oshima, J., Hasegawa, S., Iso, Y. & Tomita, Y. Supplementary document for red-sensitive organic nanoparticle-polymer composite materials for volume holographic gratings with large refractive index modulation amplitudes - 5040792.pdf, 10.6084/m9.figshare.13634240.v2 (2021).

[18] Uchida, N. Calculation of diffraction efficiency in hologram gratings attenuated along the direction perpendicular to the grating vector. *J. Opt. Soc. Am.* **63**, 280–287. 10.1364/JOSA.63.000280 (1973).

[19] Sheridan, J. A comparison of diffraction theories for off-Bragg replay. *J. Mod. Opt.* **39**, 1709–1718. 10.1080/713823578 (1992).

[20] Suzuki, N. & Tomita, Y. Holographic scattering in SiO nanoparticle-dispersed photopolymer films. *Appl. Opt.* **46**, 6809–6814. 10.1364/AO.46.006809 (2007). 10.1364/ao.46.006809 17882303

[21] Toft-Petersen, R., Georgii, R., Schneider, M., Nishiki, N. & Böni, P. Characterization of pyrolytic graphite with cold neutrons. *Nucl. Instruments Methods Phys. Res. Sect. A: Accel. Spectrometers, Detect. Assoc. Equip.* **977**, 10.1016/j.nima.2020.164341 (2020).

[22] Hadden, E. *Polymer based photonic materials for cold neutron optics*. Dissertation, Universit t Wien, Wien (2024). 10.25365/thesis.77766.

[23] Klepp, J. et al. Advancing data analysis for reflectivity measurements of holographic nanocomposite gratings. *J. Physics: Conf. Ser.* **746**, 10.1088/1742-6596/746/1/012022 (2016).

[24] Fally, M. et al. Experimental determination of nanocomposite grating structures by light- and neutron-diffraction in the multi-wave-coupling regime. *Opt. Express* **29**, 16153–16163. 10.1364/OE.424233 (2021). 34154183 10.1364/OE.424233

